# Supplementary material for: Water-assisted hydrogen spillover in Pt nanoparticle-based metal–organic framework composites
Source: Nat Commun. 2023 Sep 20;14:5836. doi: 10.1038/s41467-023-40697-w (PMC10511639; doi:10.1038/s41467-023-40697-w)
Supplement: Supplementary file 1 — Supplementary Information [file 41467_2023_40697_MOESM1_ESM.pdf]

Supplementary Information

Water-assisted hydrogen spillover in Pt nanoparticle-based metal–organic framework composites

Zhida Gu, Mengke Li, Cheng Chen, Xinglong Zhang, Chengyang Luo, Yutao Yin, Ruifa Su, Suoying Zhang, Yu Shen, Yu Fu*, Weina Zhang* & Fengwei Huo*

*Corresponding author. Email: fuyu@mail.neu.edu.cn; iamwnzhang@njtech.edu.cn; iamfwhuo@njtech.edu.cn

This Supplementary Information file contains a detailed description of the structure and stability of MOF-801, hydrogen spillover in MOF-801, region measuring of hydrogen spillover in MOF-801, mechanism analysis, structural characterization of several other MOFs and COFs, and catalytic properties of hydrogen spillover in Pt@ZIF-8, which are shown in Supplementary Figure 1-44, Supplementary Table 1 and 2, and Supplementary Discussion.

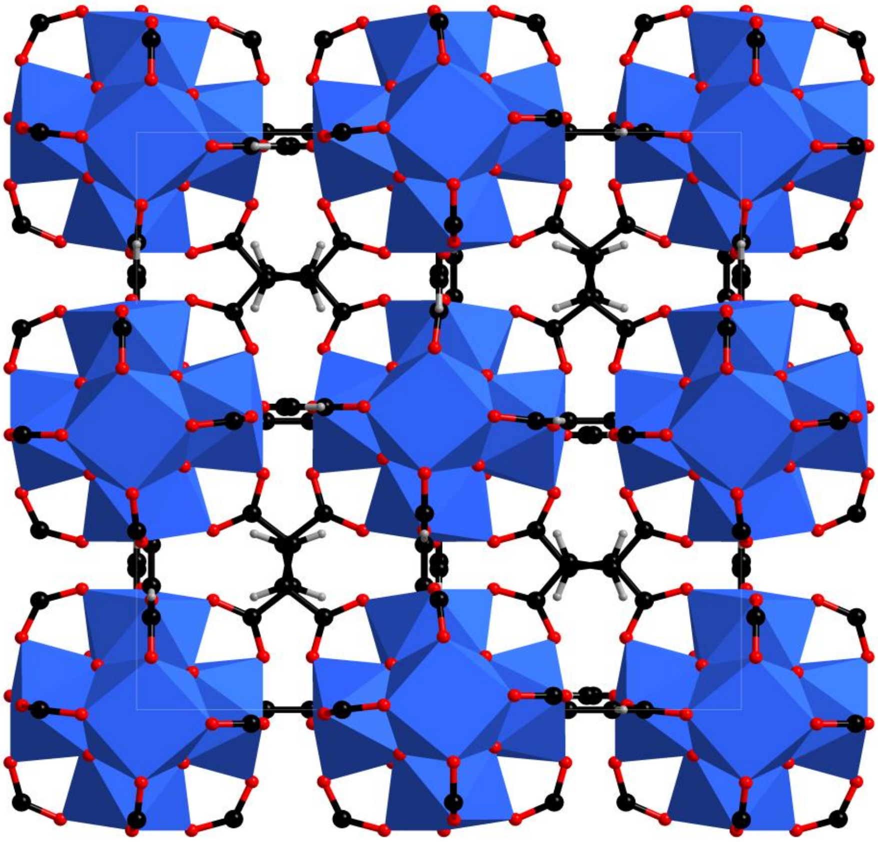

Supplementary Figure 1. Schematic diagram of MOF-801 crystal structure.

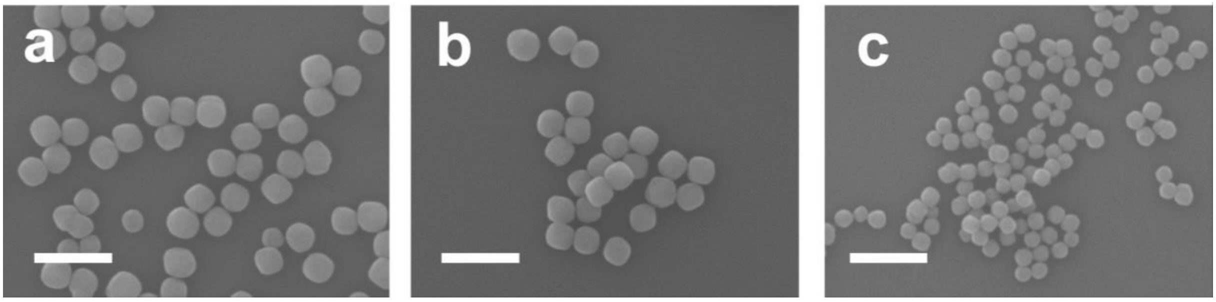

Supplementary Figure 2. SEM images of (a) MOF-801, (b) Pt@150 nm MOF-801 and (c) Pt@110 nm MOF-801. All scale bars in the images are 500 nm.

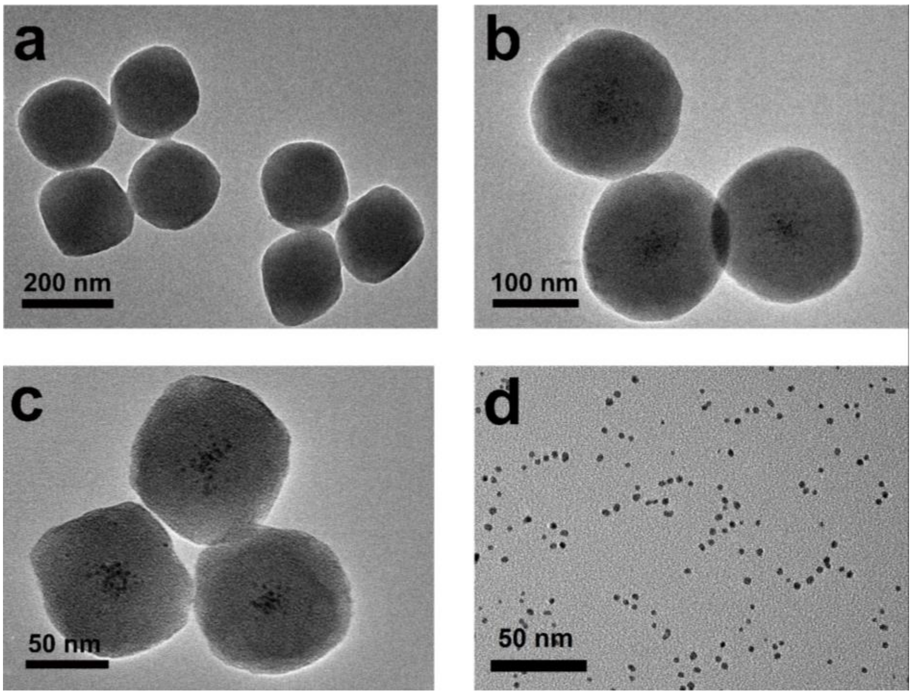

Supplementary Figure 3. TEM images of (a) MOF-801, (b) Pt@150 nm MOF-801, (c) Pt@110 nm MOF-801, (d) Pt nanoparticles.

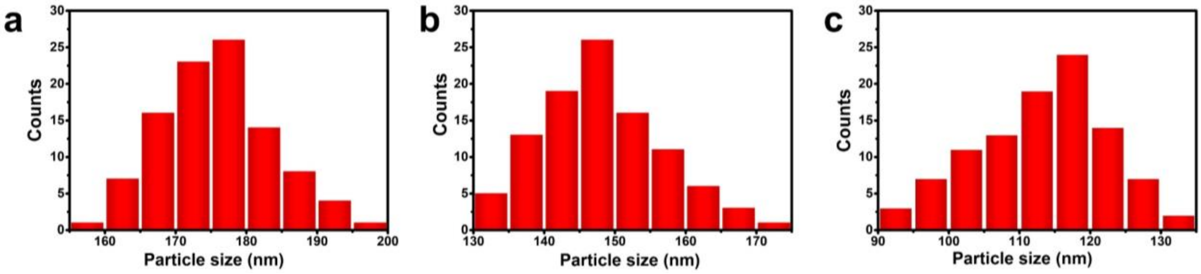

Supplementary Figure 4. Statistical size analysis of (a) MOF-801, (b) Pt@150 nm MOF-801 and (c) Pt@110 nm MOF-801 with 100 particles from TEM images.

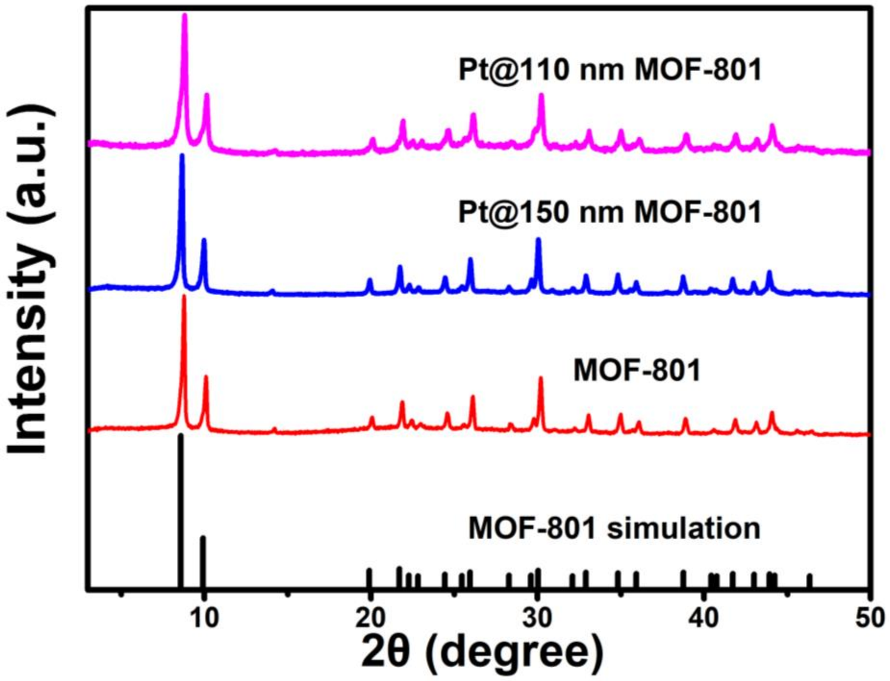

Supplementary Figure 5. PXRD patterns of MOF-801 simulation, MOF-801, Pt@150 nm MOF-801 and Pt@110 nm MOF-801.

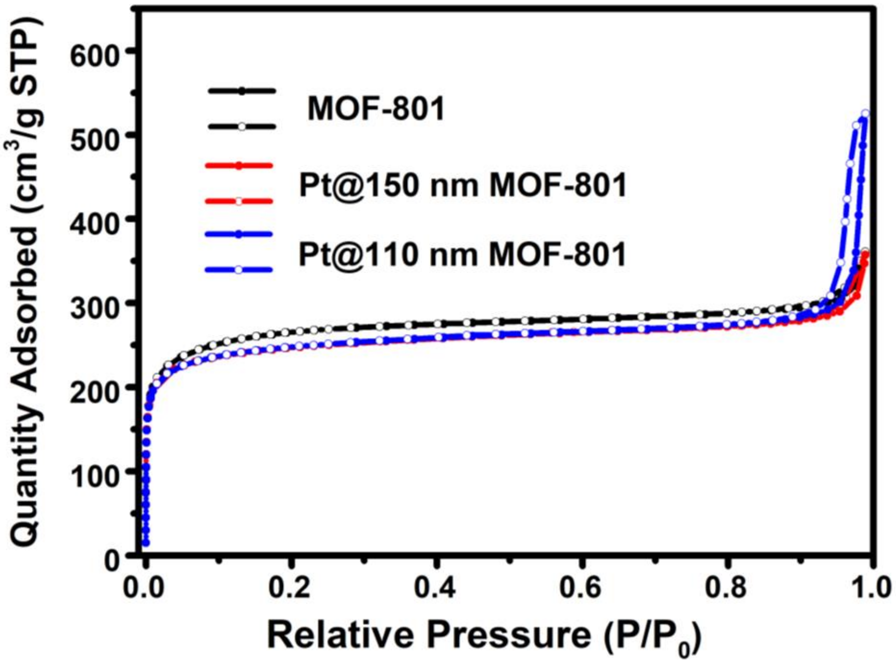

Supplementary Figure 6. Nitrogen adsorption–desorption isotherms of MOF-801, Pt@150 nm MOF-801 and Pt@110 nm MOF-801.

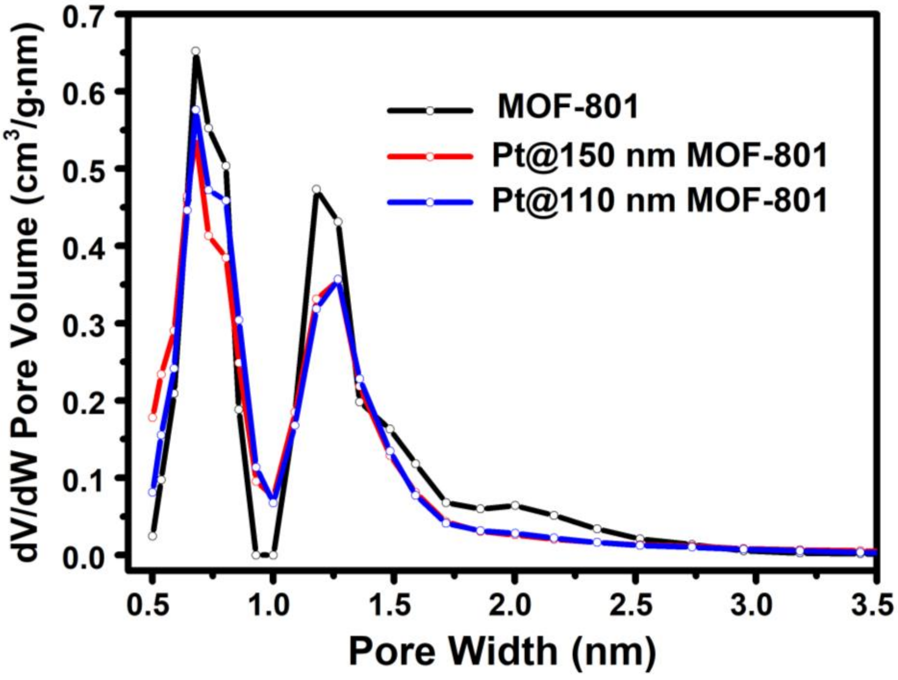

Supplementary Figure 7. Pore size distribution of MOF-801, Pt@150 nm MOF-801 and Pt@110 nm MOF-801.

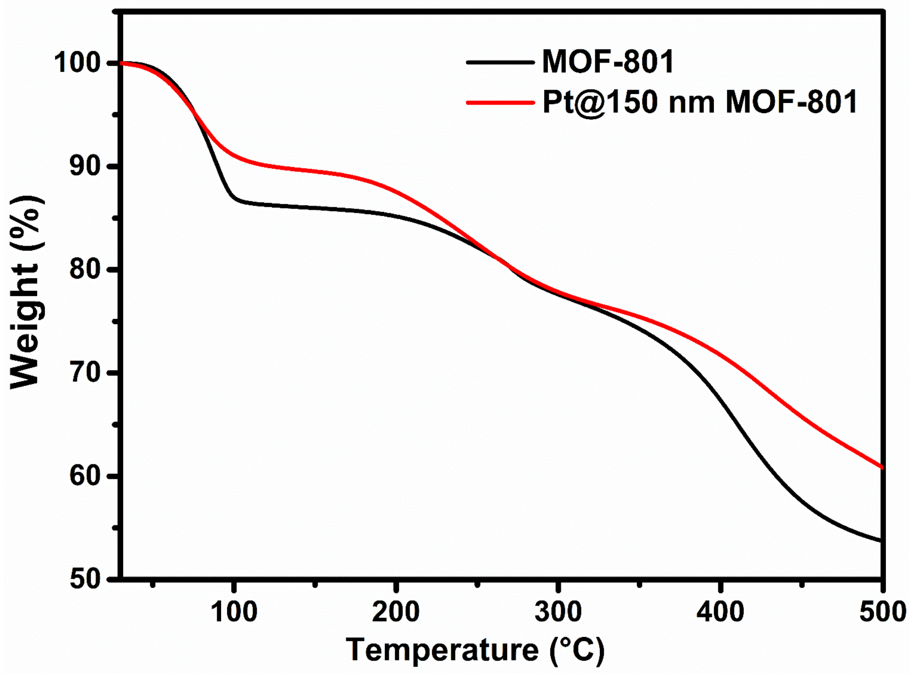

Supplementary Figure 8. TGA curves of MOF-801 and Pt@150 nm MOF-801 in nitrogen.

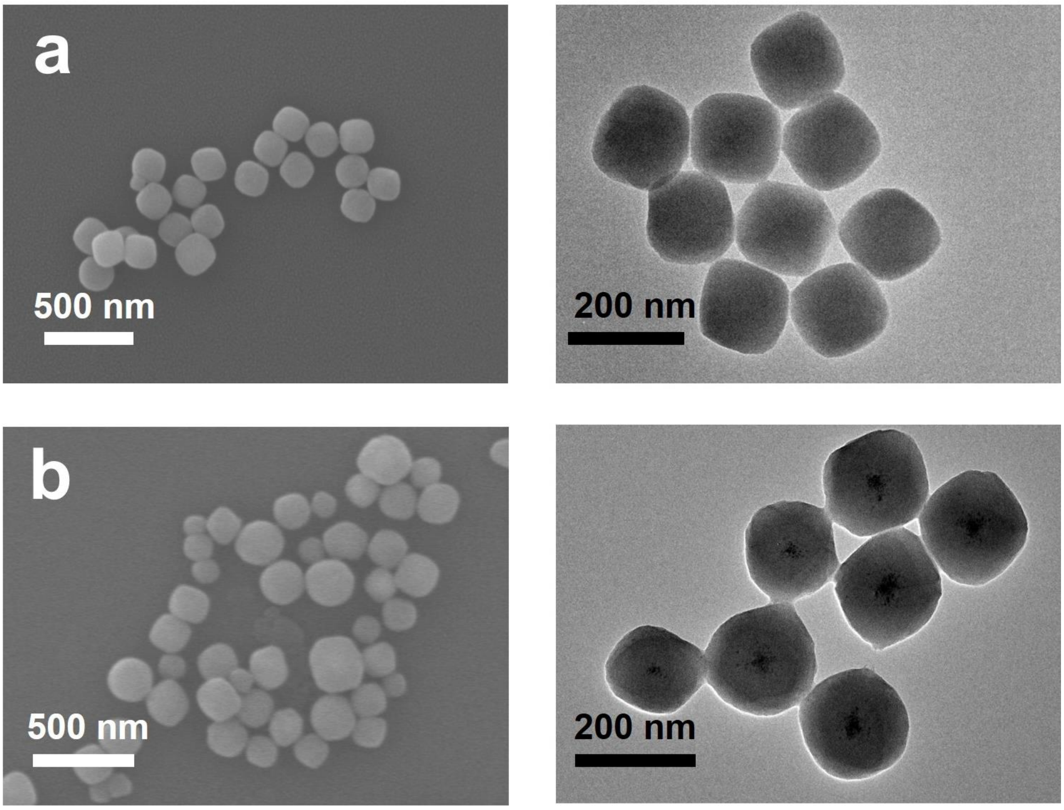

Supplementary Figure 9. SEM and TEM images of (a) MOF-801 in wet H_2 at 200 °C and (b) Pt@150 nm MOF-801 in wet N_2 at 200 °C.

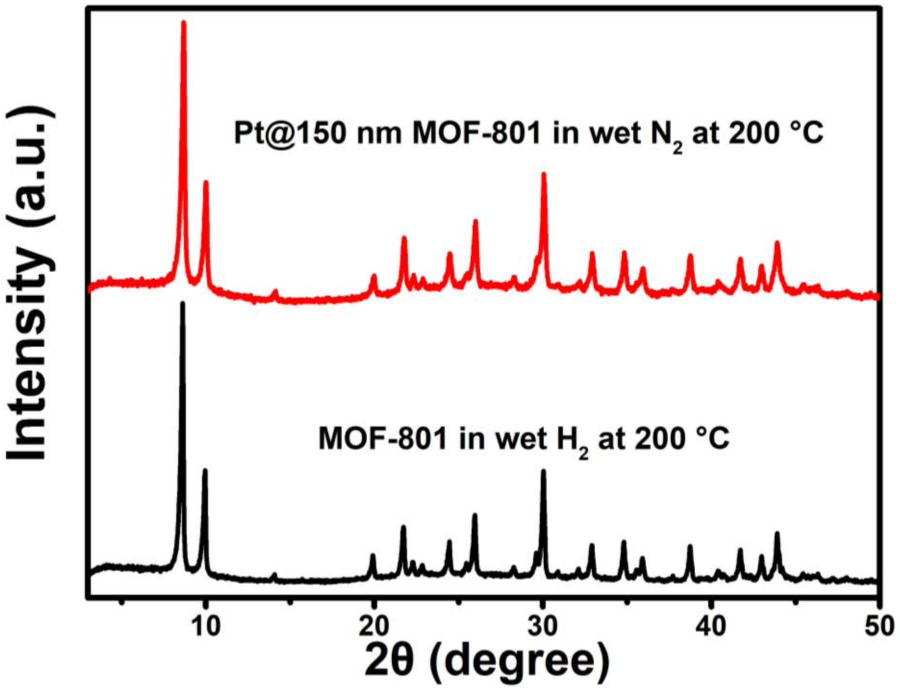

Supplementary Figure 10. PXRD patterns of MOF-801 in wet H_2 at 200 °C and Pt@150 nm MOF-801 in wet N_2 at 200 °C.

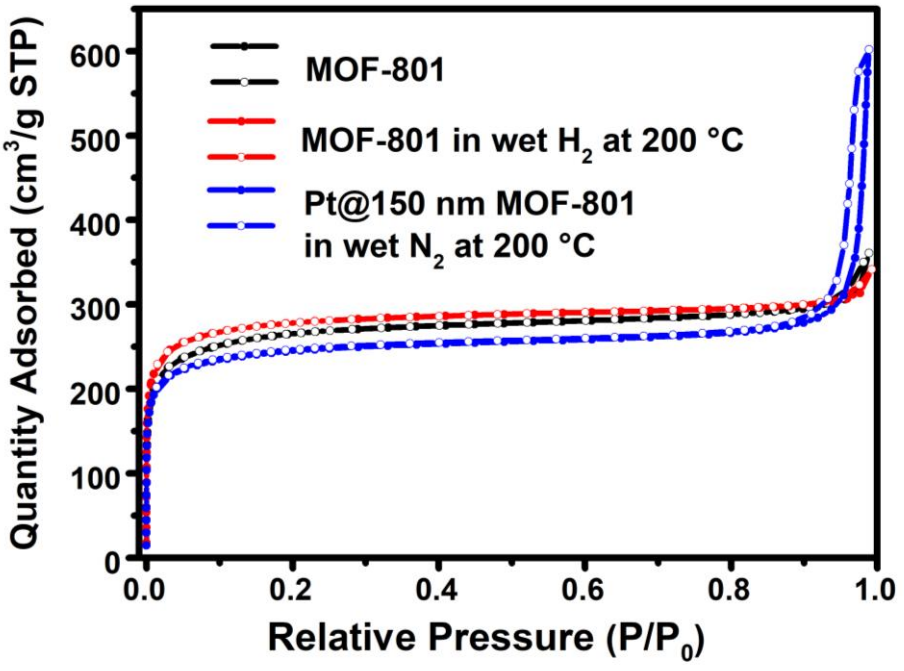

Supplementary Figure 11. Nitrogen adsorption–desorption isotherms of MOF-801, MOF-801 in wet H_2 at 200 °C and Pt@150 nm MOF-801 in wet N_2 at 200 °C.

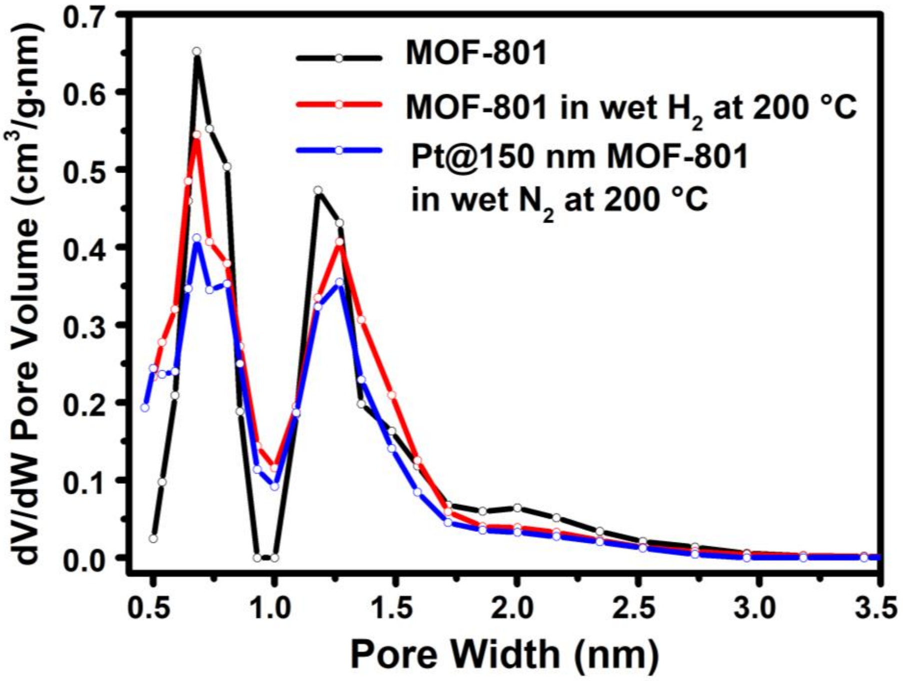

Supplementary Figure 12. Pore size distribution of MOF-801, MOF-801 in wet H_2 at 200 °C and Pt@150 nm MOF-801 in wet N_2 at 200 °C.

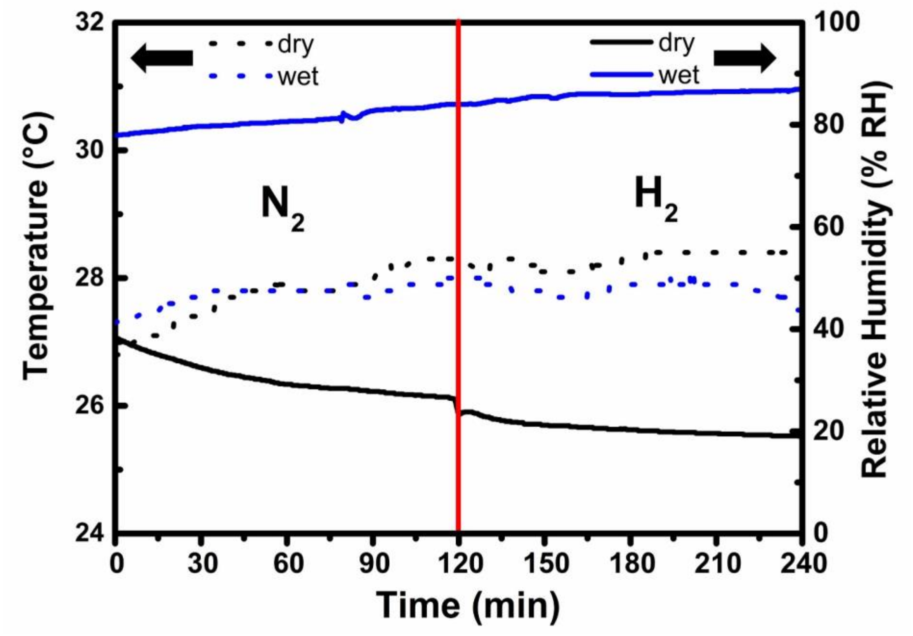

Supplementary Figure 13. Temperature and humidity of the input H_2.

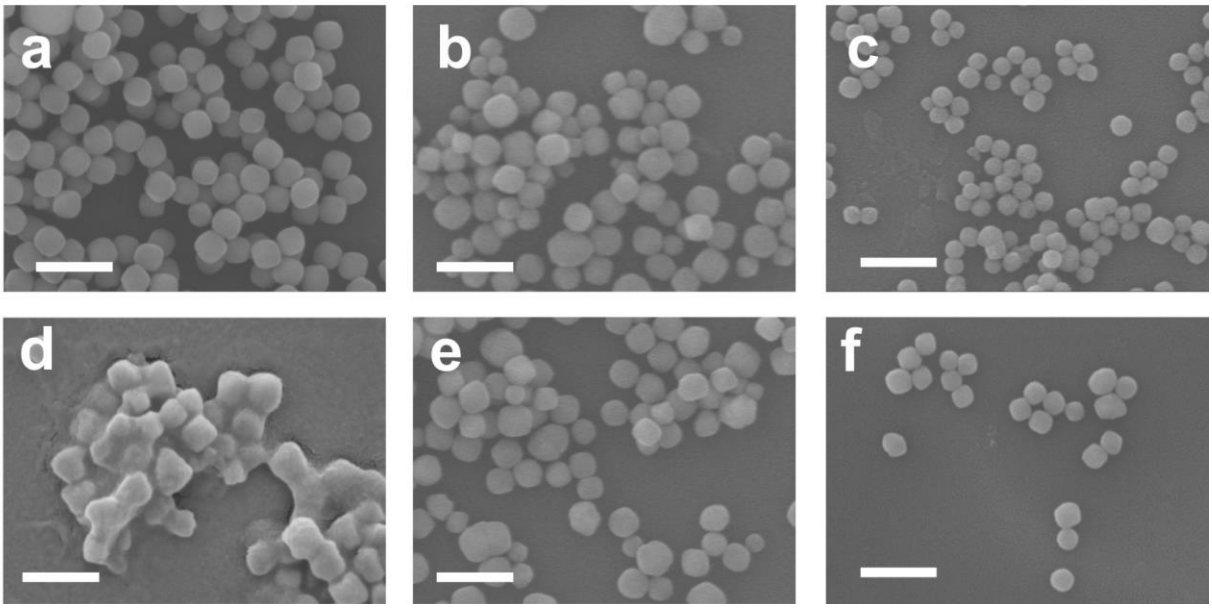

Supplementary Figure 14. SEM images of (a) Pt-MOF-801(dry), (b) Pt@150 nm MOF-801(dry), (c) Pt@110 nm MOF-801(dry), (d) Pt-MOF-801(wet), (e) Pt@150 nm MOF-801(wet), and (f) Pt@110 nm MOF-801(wet). All scale bars in the images are 500 nm.

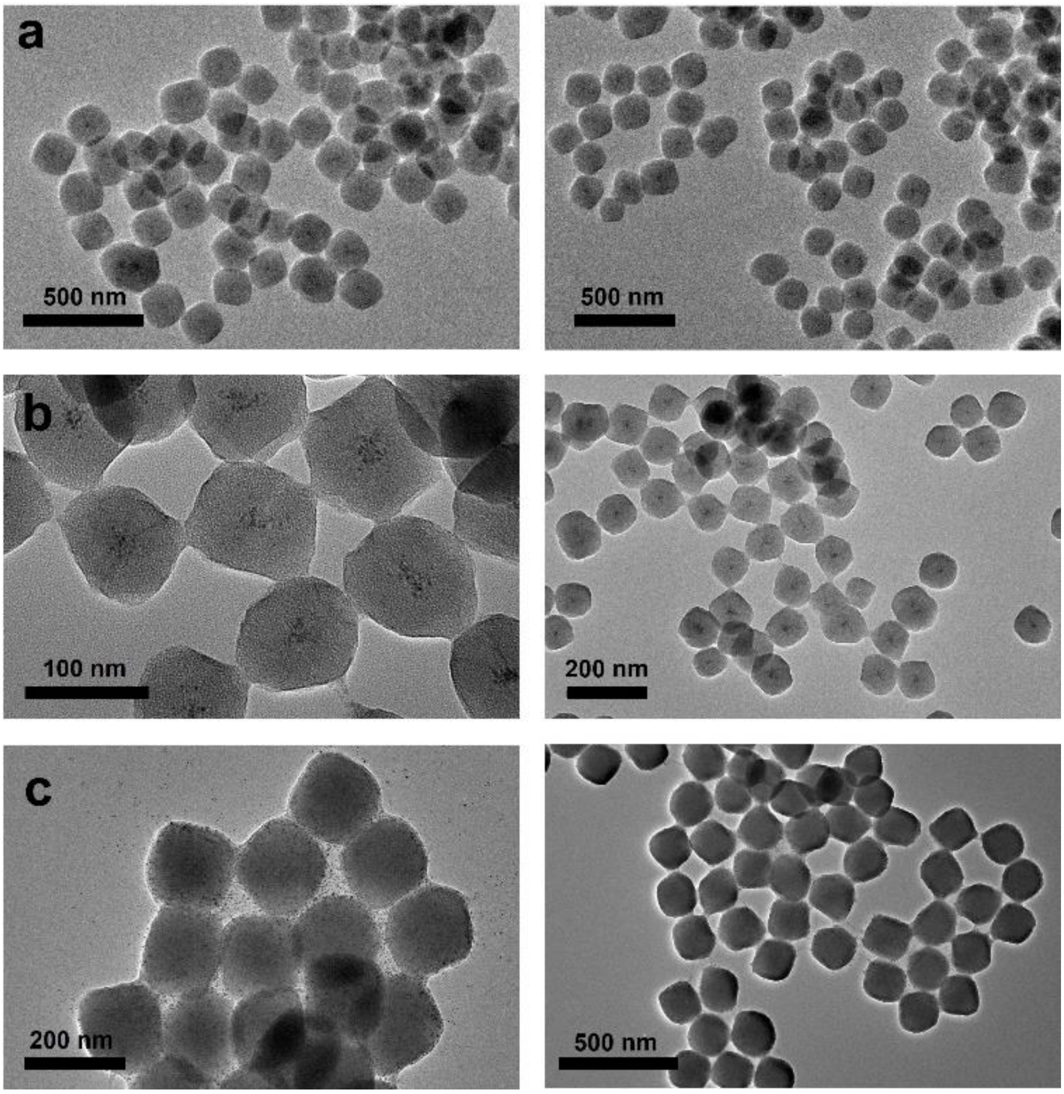

Supplementary Figure 15. TEM images of (a) Pt@150 nm MOF-801(wet), (b) Pt@110 nm MOF-801(wet) and (c) Pt-MOF-801(wet).

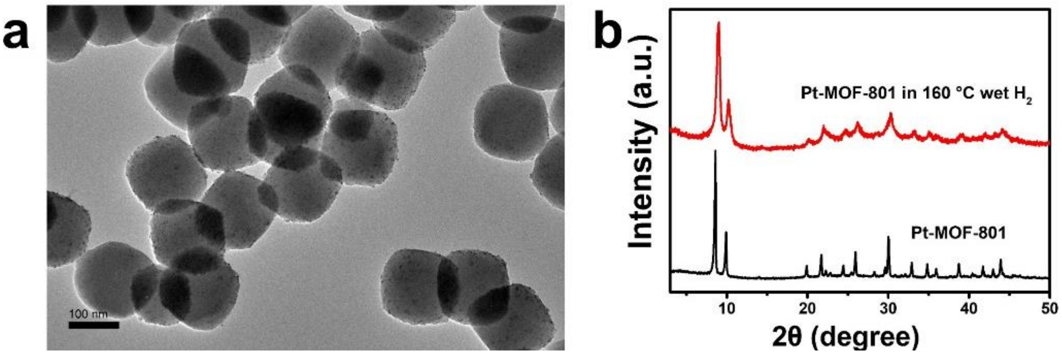

Supplementary Figure 16. (a) TEM image and (b) XRD pattern of Pt-MOF-801 in 160 °C wet H_2.

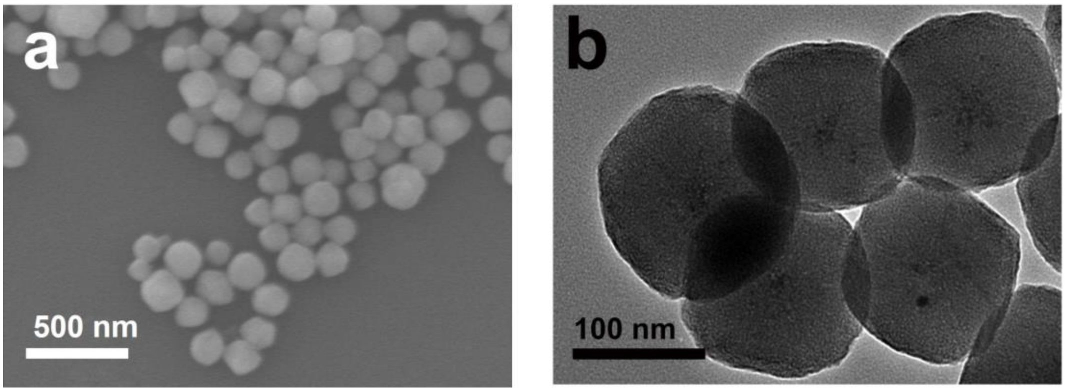

Supplementary Figure 17. SEM and TEM images of Pt@150 nm MOF-801 in H_2 at 300 °C.

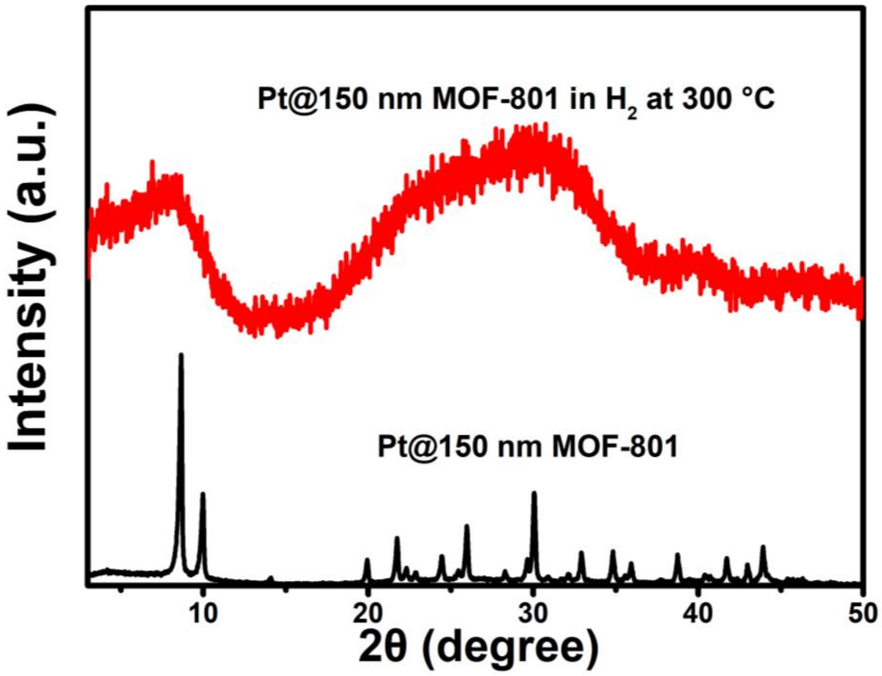

Supplementary Figure 18. PXRD patterns of Pt@150 nm MOF-801 and Pt@150 nm MOF-801 in H_2 at 300 °C.

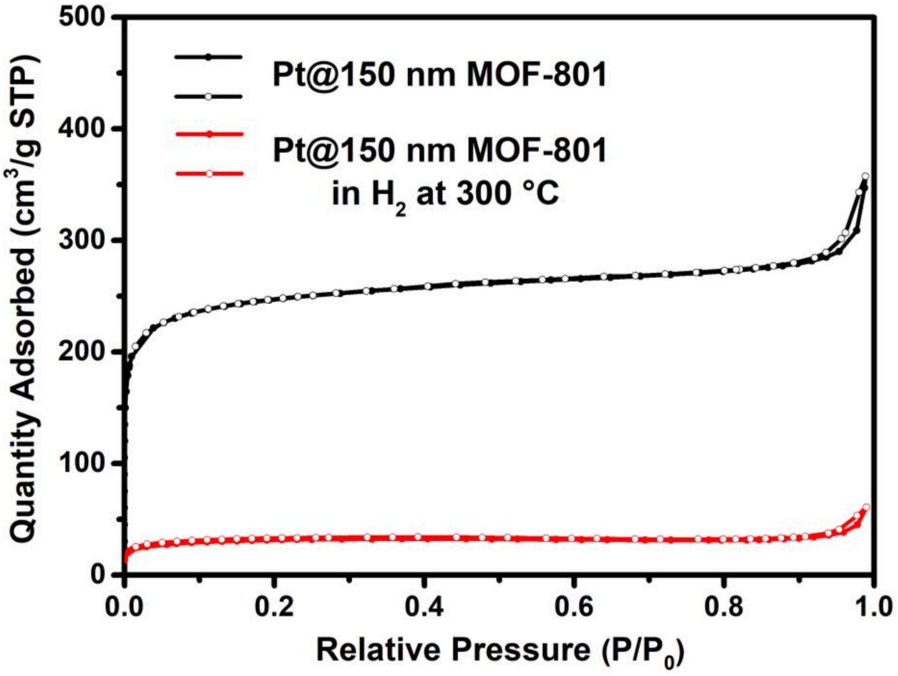

Supplementary Figure 19. Nitrogen adsorption–desorption isotherms of Pt@150 nm MOF-801 and Pt@150 nm MOF-801 in H_2 at 300 °C.

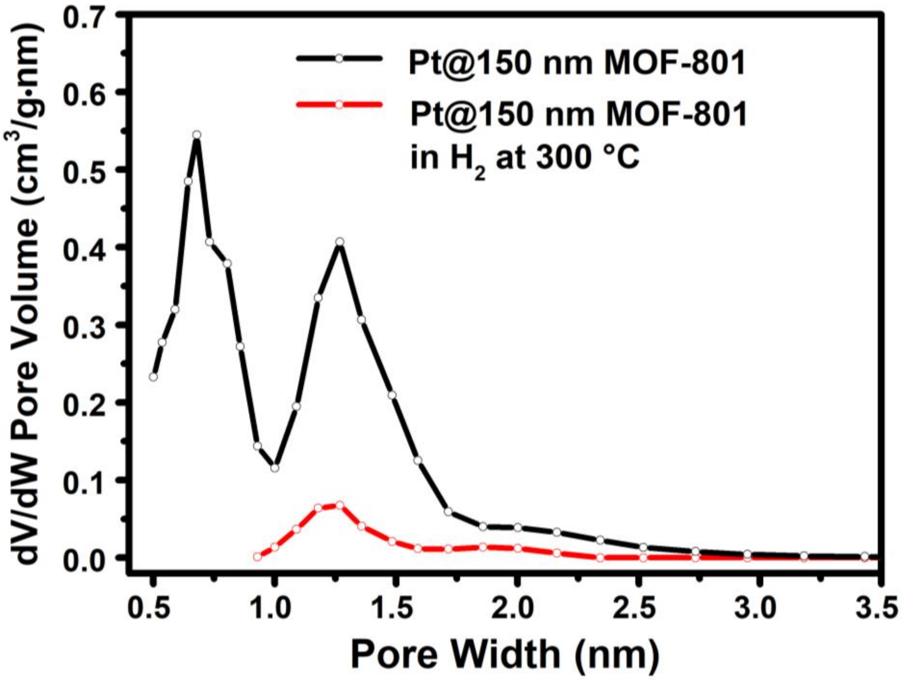

Supplementary Figure 20. Pore size distribution of Pt@150 nm MOF-801 and Pt@150 nm MOF-801 in H_2 at 300 °C.

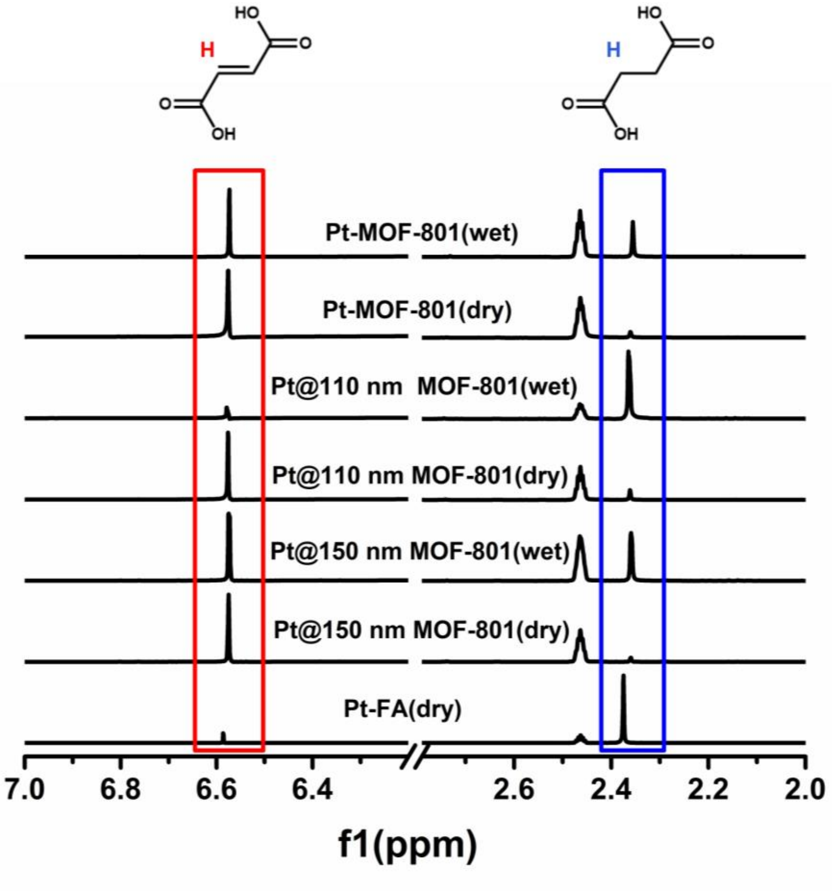

Supplementary Figure 21. NMR curves of Pt-FA(dry), Pt@150 nm MOF-801(dry), Pt@150 nm MOF-801(wet), Pt@110 nm MOF-801(dry), Pt@110 nm MOF-801(wet), Pt-MOF-801(dry), and Pt-MOF-801(wet).

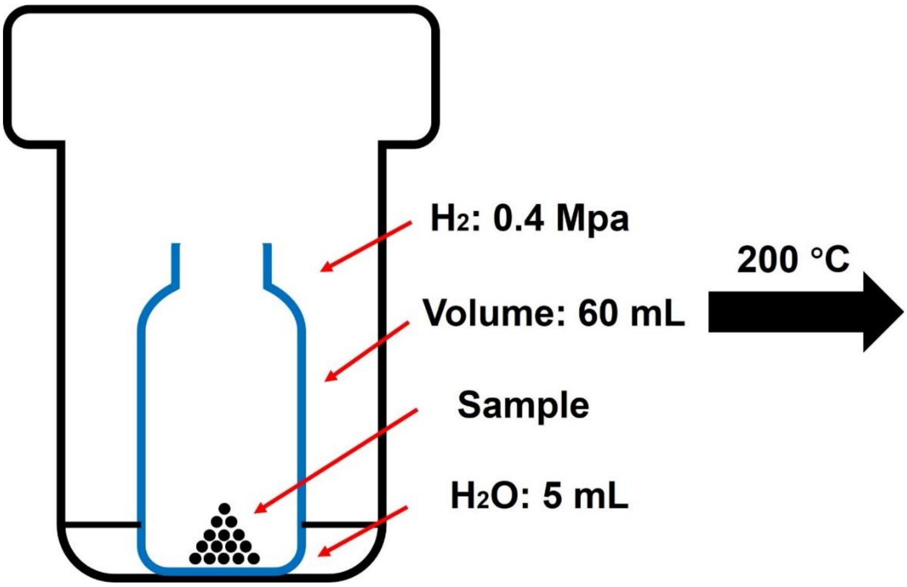

Supplementary Figure 22. Schematic diagram of the reactor for further improving the hydrogen spillover efficiency under higher H_2 pressure and humidity.

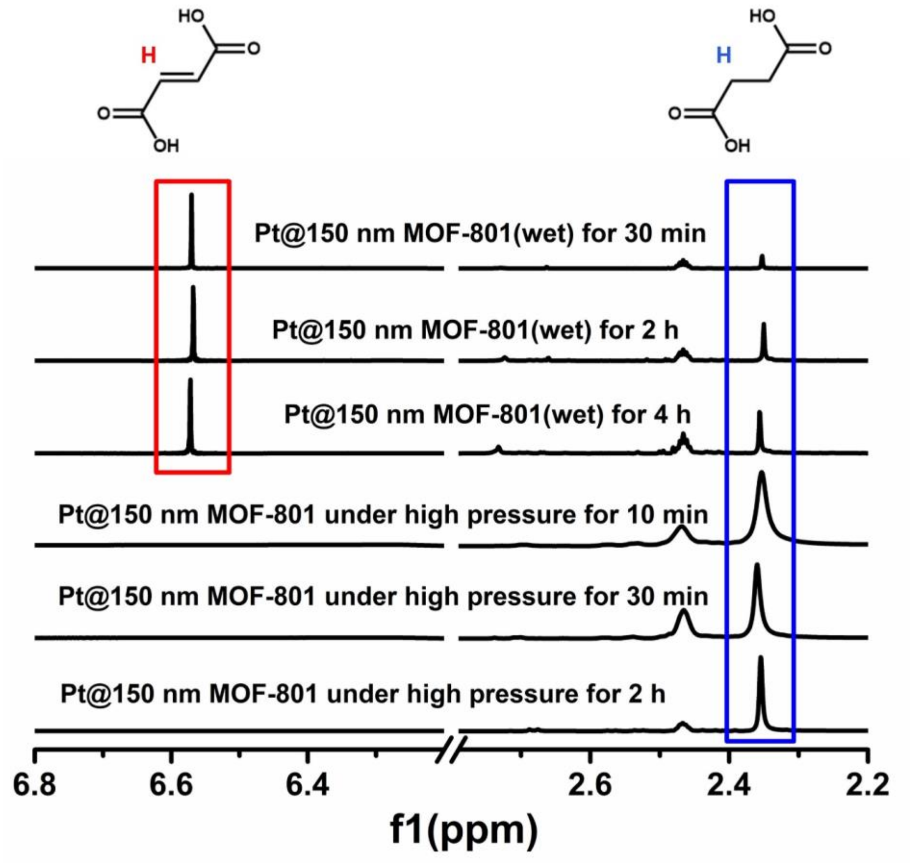

Supplementary Figure 23. NMR curves of Pt@150 nm MOF-801 under high pressure for 2 h, Pt@150 nm MOF-801 under high pressure for 30 min, Pt@150 nm MOF-801 under high pressure for 10 min, Pt@150 nm MOF-801(wet) for 4 h, Pt@150 nm MOF-801(wet) for 2 h, and Pt@150 nm MOF-801(wet) for 30 min.

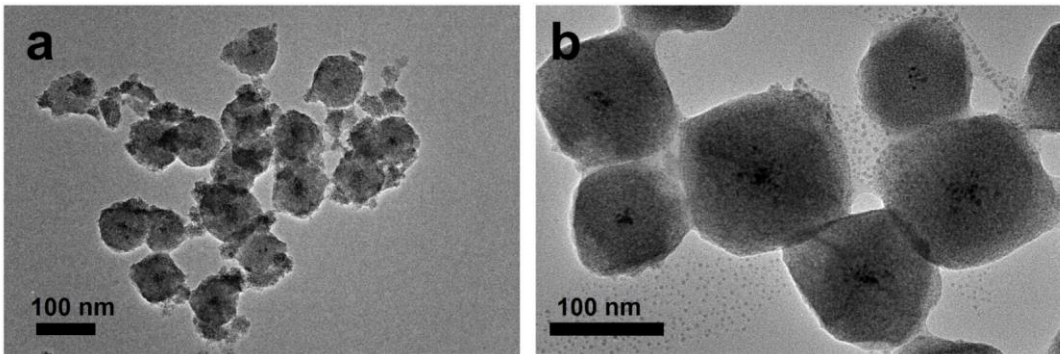

Supplementary Figure 24. TEM images of (a) Pt@110 nm MOF-801 under high pressure, and (b) Pt@150 nm MOF-801 under high pressure.

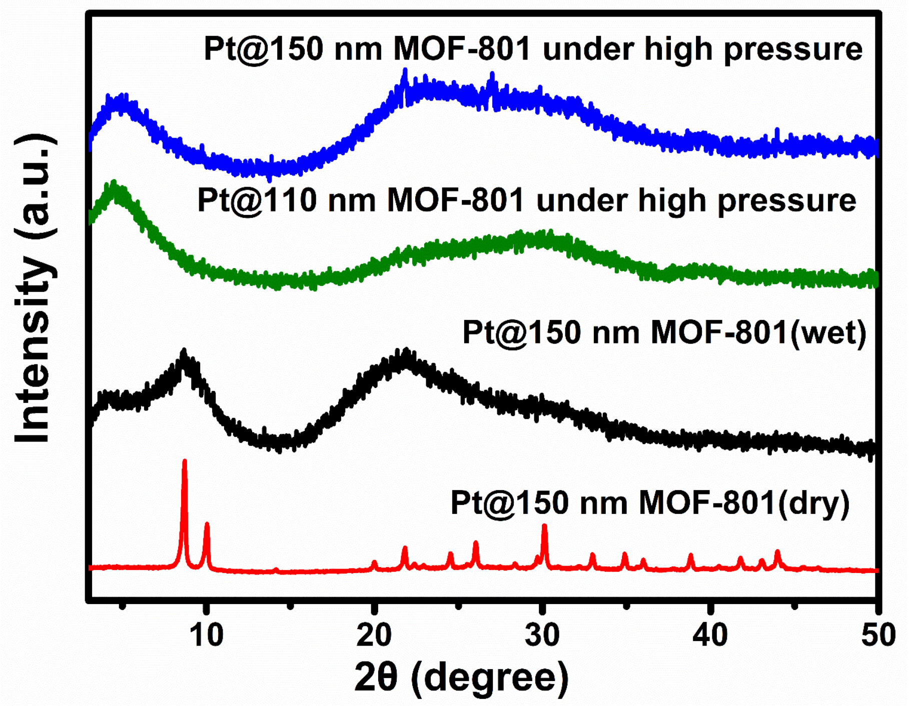

Supplementary Figure 25. PXRD patterns of Pt@150 nm MOF-801(dry), Pt@150 nm MOF-801(wet), Pt@110 nm MOF-801 under high pressure, and Pt@150 nm MOF-801 under high pressure.

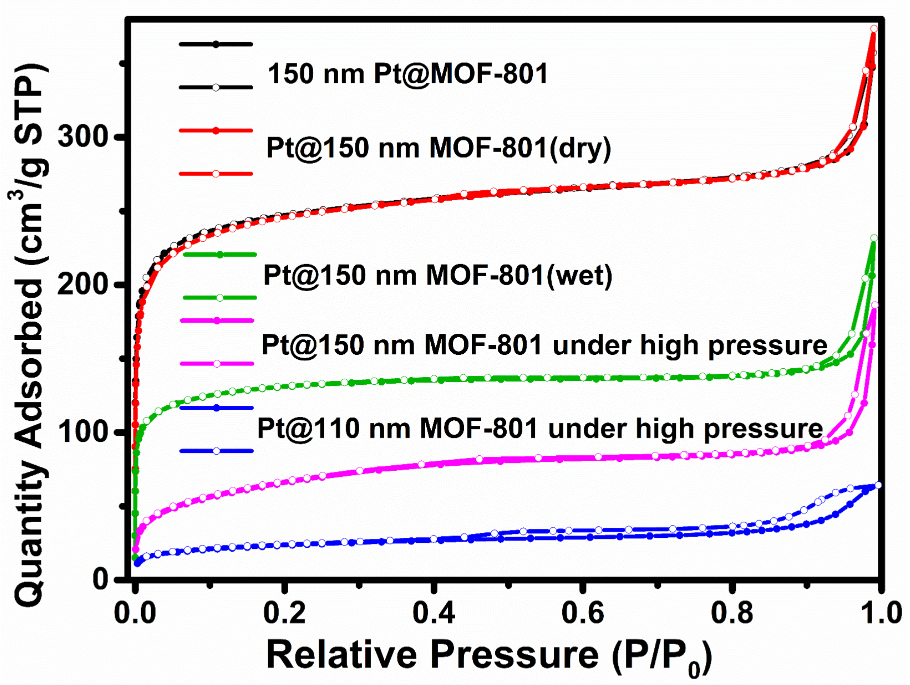

Supplementary Figure 26. Nitrogen adsorption–desorption isotherms of Pt@150 nm MOF-801(dry), Pt@150 nm MOF-801(wet), Pt@150 nm MOF-801 under high pressure, and Pt@110 nm MOF-801 under high pressure.

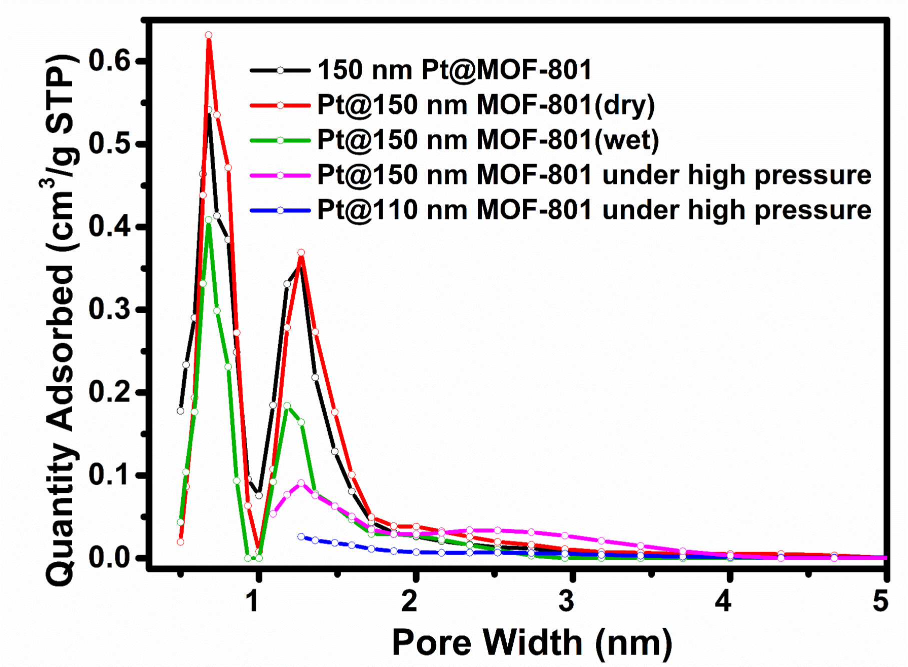

Supplementary Figure 27. Pore size distribution of Pt@150 nm MOF-801, Pt@150 nm MOF-801(dry), Pt@150 nm MOF-801(wet), Pt@150 nm MOF-801 under high pressure, and Pt@110 nm MOF-801 under high pressure.

Supplementary Discussion. Hydrogen spillover under high H_2 pressure and humidity.

After thermal treatment at 200 °C for 2 h, the Pt@110 nm MOF-801 and the Pt@150 nm MOF-801 exhibited morphology damage to MOF particles (Supplementary Fig. 24). Moreover, after such treatment, both samples lost all the characteristic peaks of MOF-801 and presented more grievous crystallographic structural collapse according to the PXRD pattern (Supplementary Fig. 25). In addition, the significant decrease in BET surface area (Supplementary Fig. 26) and pore volume (Supplementary Fig. 27) all indicated the complete destruction of the MOF structure due to the hydrogen spillover. Furthermore, both samples obtained nearly 100% ligand conversion after treatment (Supplementary Fig. 23), indicating that the efficiency of hydrogen spillover can be regulated with H_2 pressure and humidity. Particularly, the nearly 100% ligand conversion (Supplementary Fig. 23) and the obvious adhesion phenomenon (Supplementary Fig. 24b) of Pt@150 nm MOF-801 under high pressure suggested a larger spillover region, which essentially spanned around 150 nm in diameter of the whole MOF particles. It's important to take notice that the spillover region, which has a diameter of 100 or 150 nm, refers to the coverage of hydrogen spillover rather than the spillover distance[1]. Due to the accumulation of Pt nanoparticles within a central range of approximately 50 nm in diameter in both Pt@110 nm MOF-801 and Pt@150 nm MOF-801, the spillover regions may be converted to spillover distances of at least 30 nm at atmospheric pressure and 50 nm at higher humidity and pressure, which is based on the distance from the accumulation edge of the Pt nanoparticles to the MOF surface.

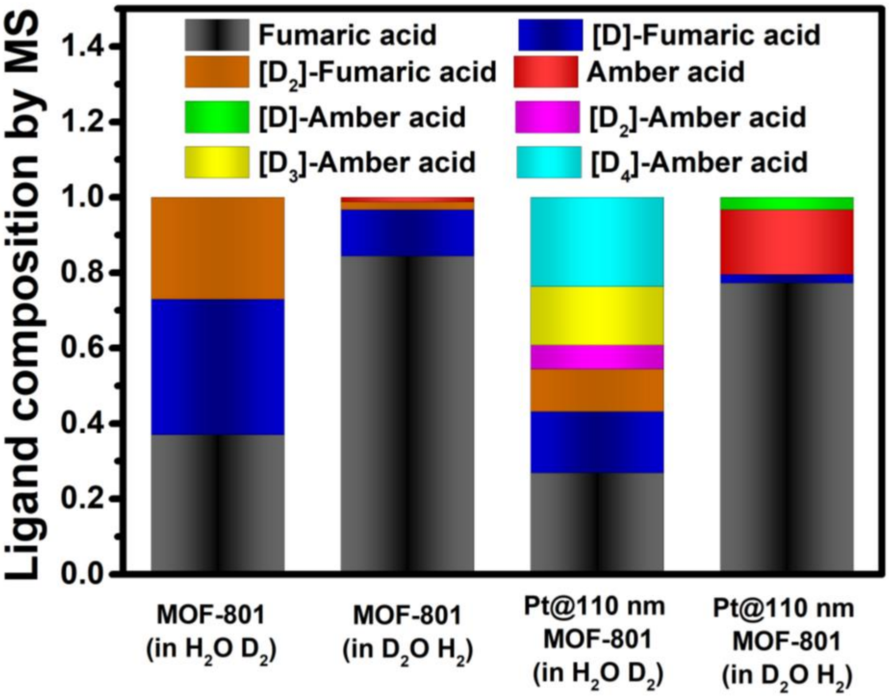

Supplementary Figure 28. Ligand composition of MOF-801 and Pt@110 nm MOF-801 in H_2O-containing D_2 or D_2O-containing H_2 respectively by MS.

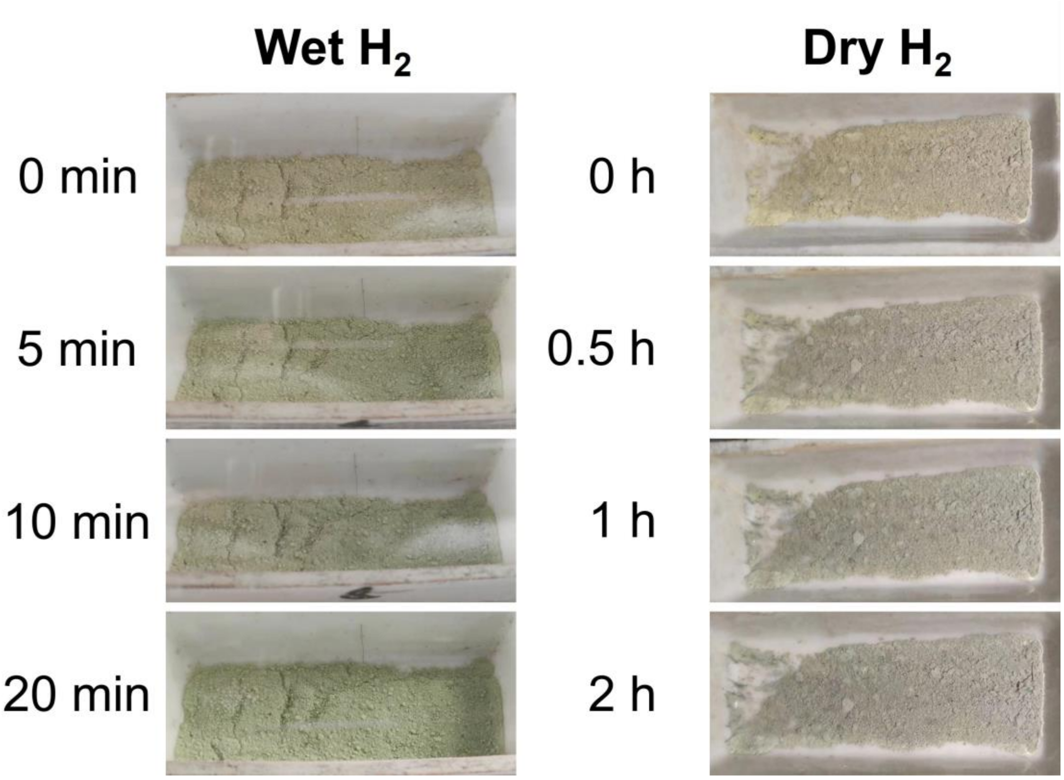

Supplementary Figure 29. WO_3 and Pt@110 nm MOF-801 in wet or dry H_2 at 200 °C for different lengths of time.

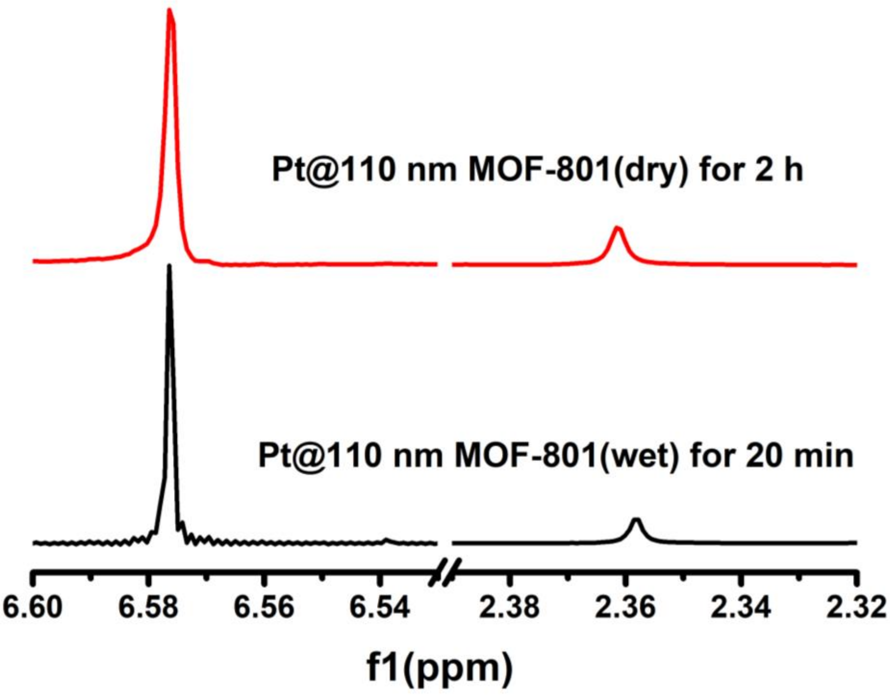

Supplementary Figure 30. NMR curves of Pt@110 nm MOF-801(dry) for 2 h and Pt@110 nm MOF-801(wet) for 20 min.

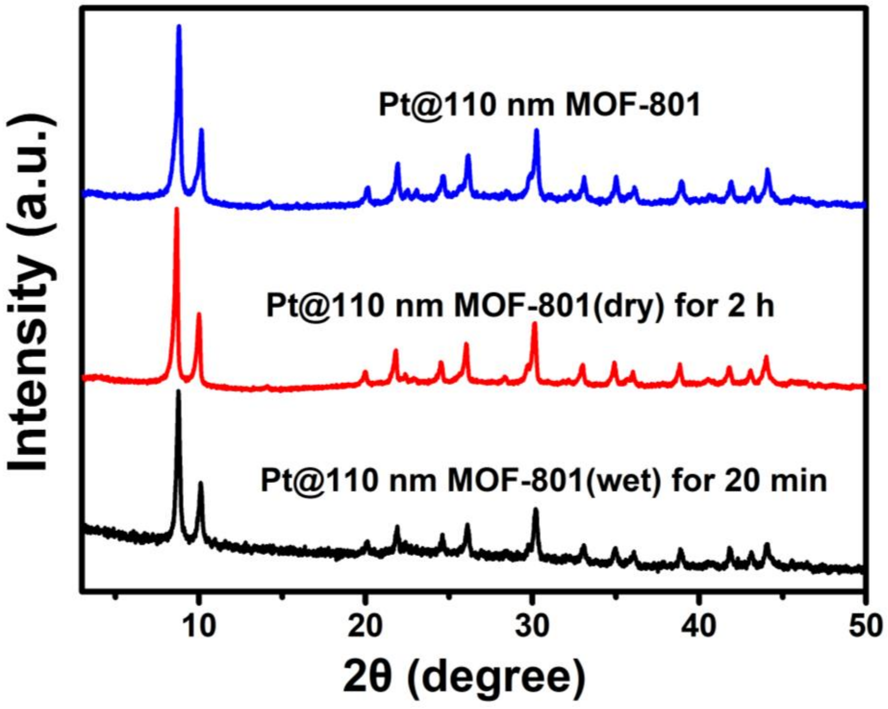

Supplementary Figure 31. PXRD patterns of Pt@110 nm MOF-801, Pt@110 nm MOF-801(dry) for 2 h and Pt@110 nm MOF-801(wet) for 20 min.

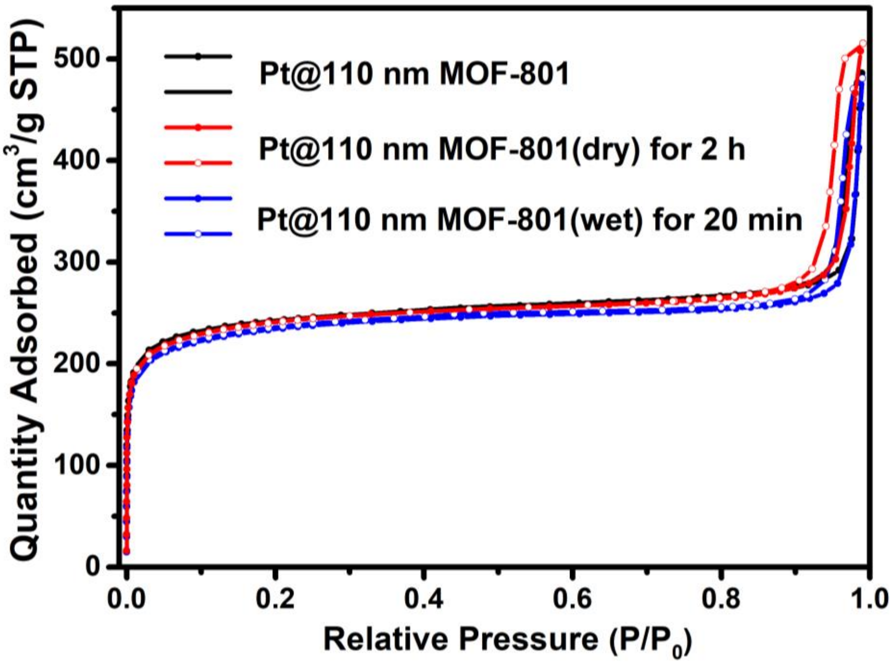

Supplementary Figure 32. Nitrogen adsorption–desorption isotherms of Pt@110 nm MOF-801, Pt@110 nm MOF-801(dry) for 2 h and Pt@110 nm MOF-801(wet) for 20 min.

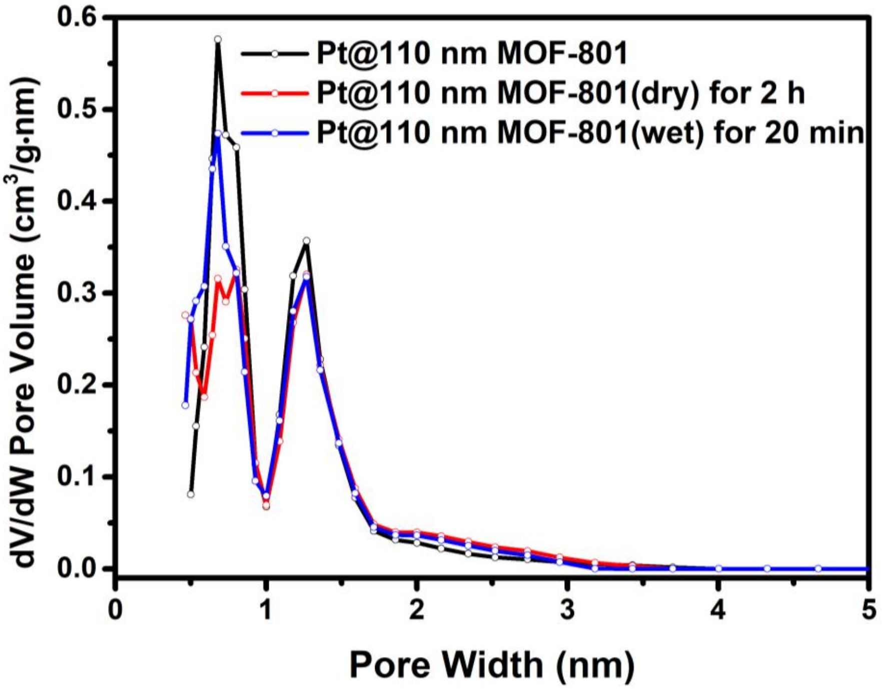

Supplementary Figure 33. Pore size distribution of Pt@110 nm MOF-801, Pt@110 nm MOF-801(dry) for 2 h and Pt@110 nm MOF-801(wet) for 20 min.

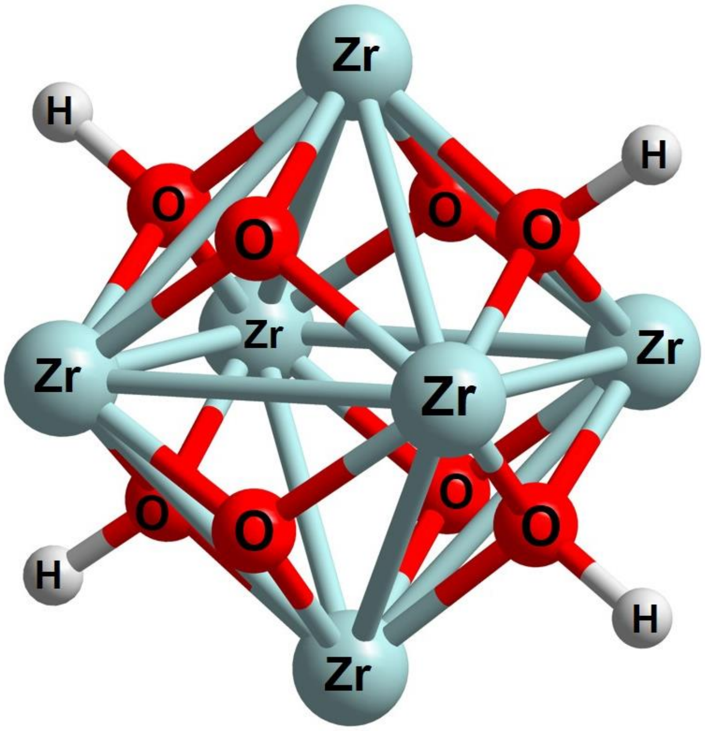

Supplementary Figure 34. Scheme of the μ_3-OH in a zirconium-oxygen cluster.

Supplementary Table 1. The calculated H migration energy barriers (Ha) along Path 1 and 2.

Path1-position (a.u.)	Path1-energy barriers (Ha)	Path1-position (a.u.)	Path2-energy barriers (Ha)
0.000000	0.000000	0.000000	0.000000
0.117526	0.095011	0.107762	0.082566
0.212791	0.144212	0.202238	0.104772
0.266034	0.149935	0.311204	0.122211
0.298601	0.151171	0.409507	0.131919
0.384830	0.144668	0.452131	0.133560
0.500000	0.107088	0.500000	0.134342
0.615171	0.144668	0.547870	0.133560
0.701399	0.151171	0.590494	0.131919
0.733967	0.149935	0.688796	0.122211
0.787209	0.144212	0.797762	0.104772
0.882475	0.095011	0.892239	0.082566
1.000000	0.000000	1.000000	0.000000

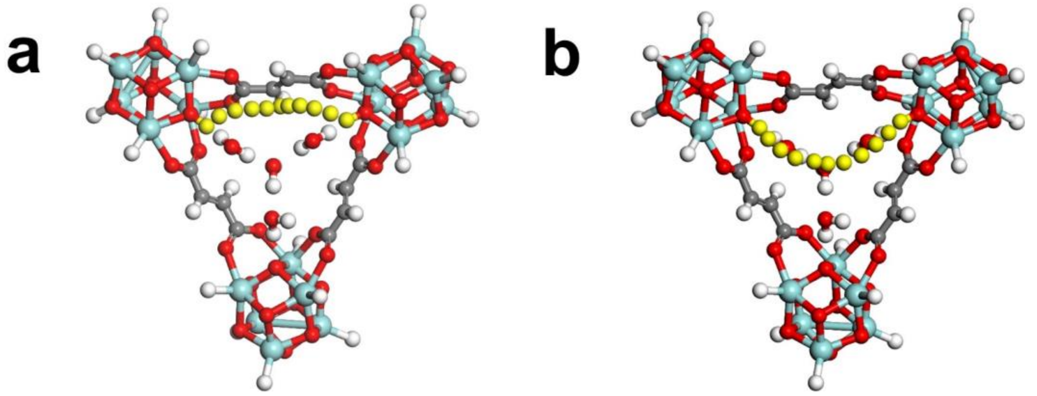

Supplementary Figure 35. Scheme of two spillover paths including (a) the first ligand spillover path and (b) the second water-assisted spillover path.

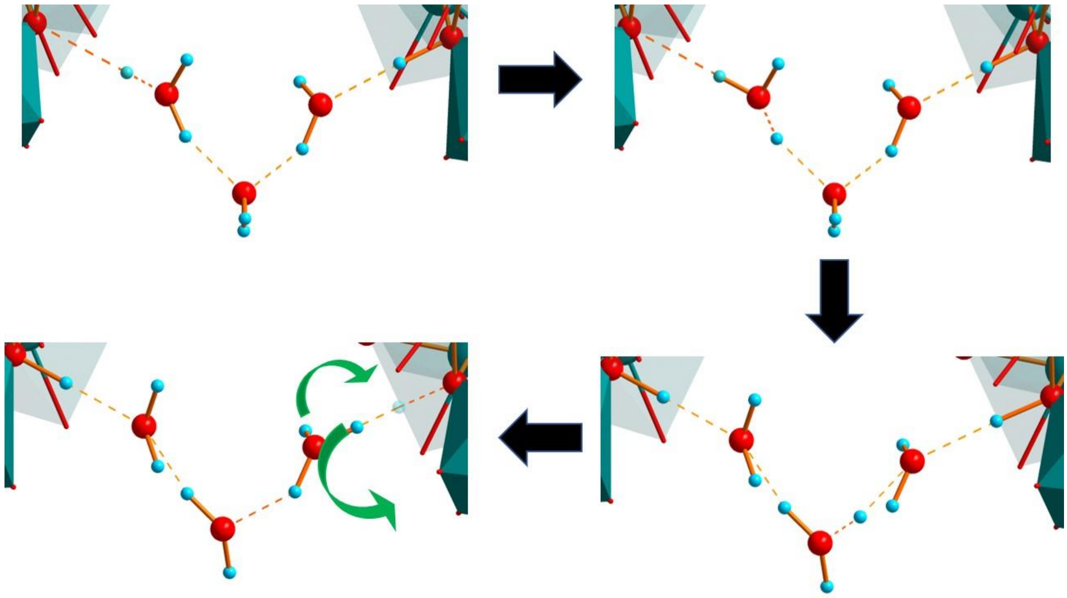

Supplementary Figure 36. Scheme of a possible path for hydrogen transfer via water.

Supplementary Table 2. The calculated hydrogenation energy on MOF-801 (unit: Ha)

	$E_{(MOF+H)}$	$E_{(MOF)}$	$E_{(H_2)}$	ΔE
MOF	−67,219.50	−67,218.83	−1.1542	−0.092

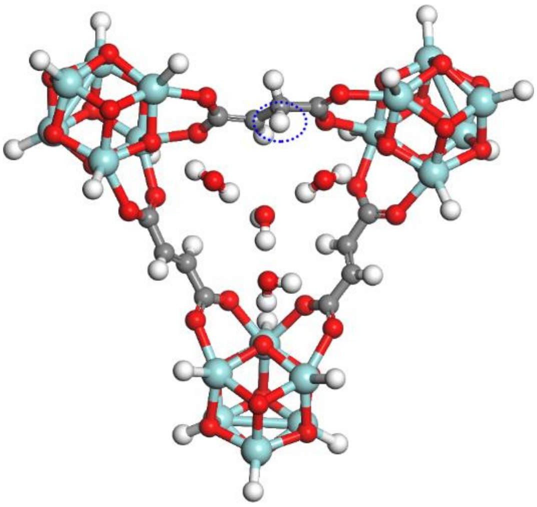

Supplementary Figure 37. The optimized structure of hydrogenation on MOF-801 at -CH-CH- site.

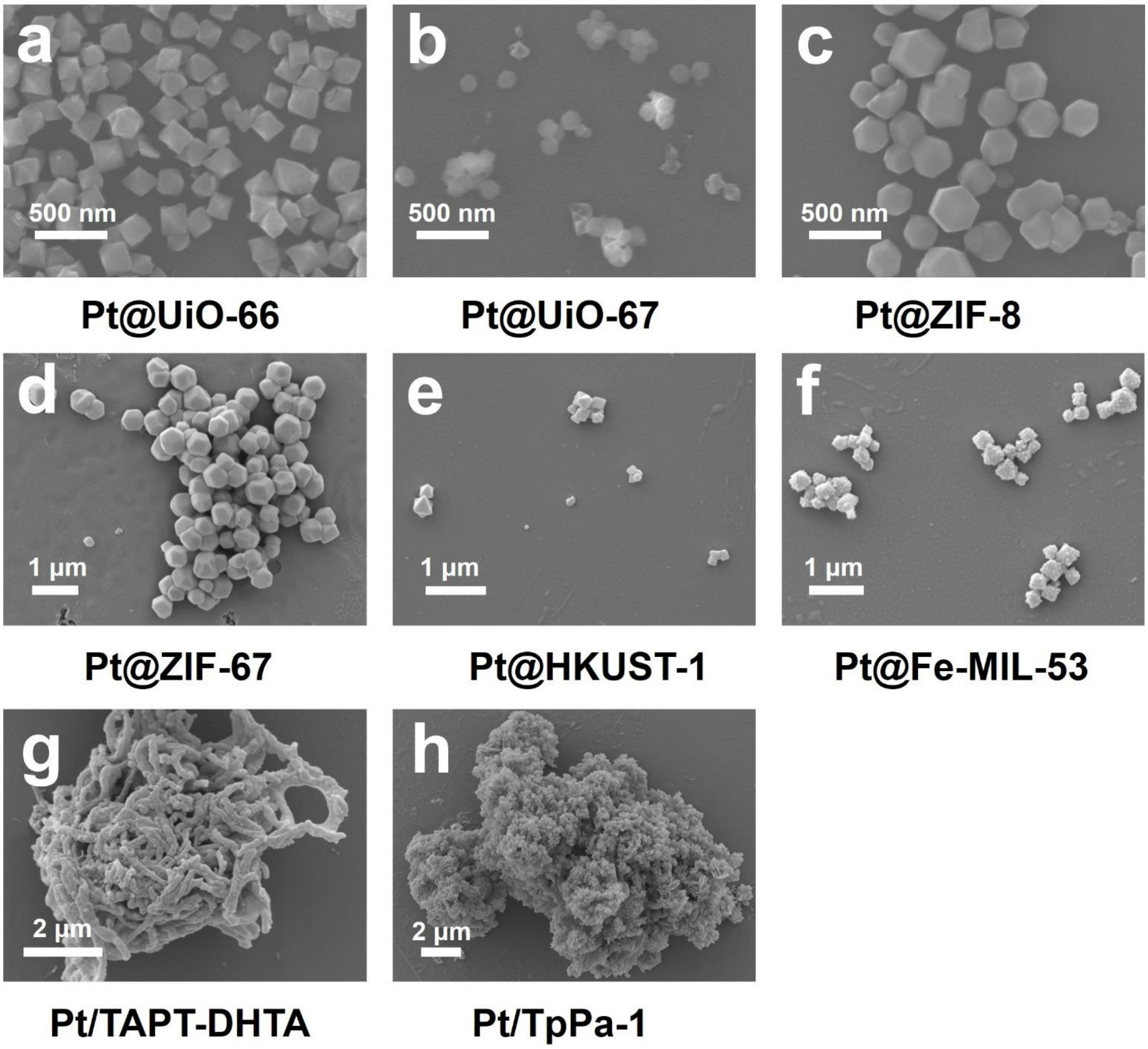

Supplementary Figure 38. SEM images of (a) Pt@UiO-66, (b) Pt@UiO-67, (c) Pt@ZIF-8, (d) Pt@ZIF-67, (e) Pt@HKUST-1, (f) Pt@Fe-MIL-53, (g) Pt/TAPT-DHTA and (h) Pt/TpPa-1.

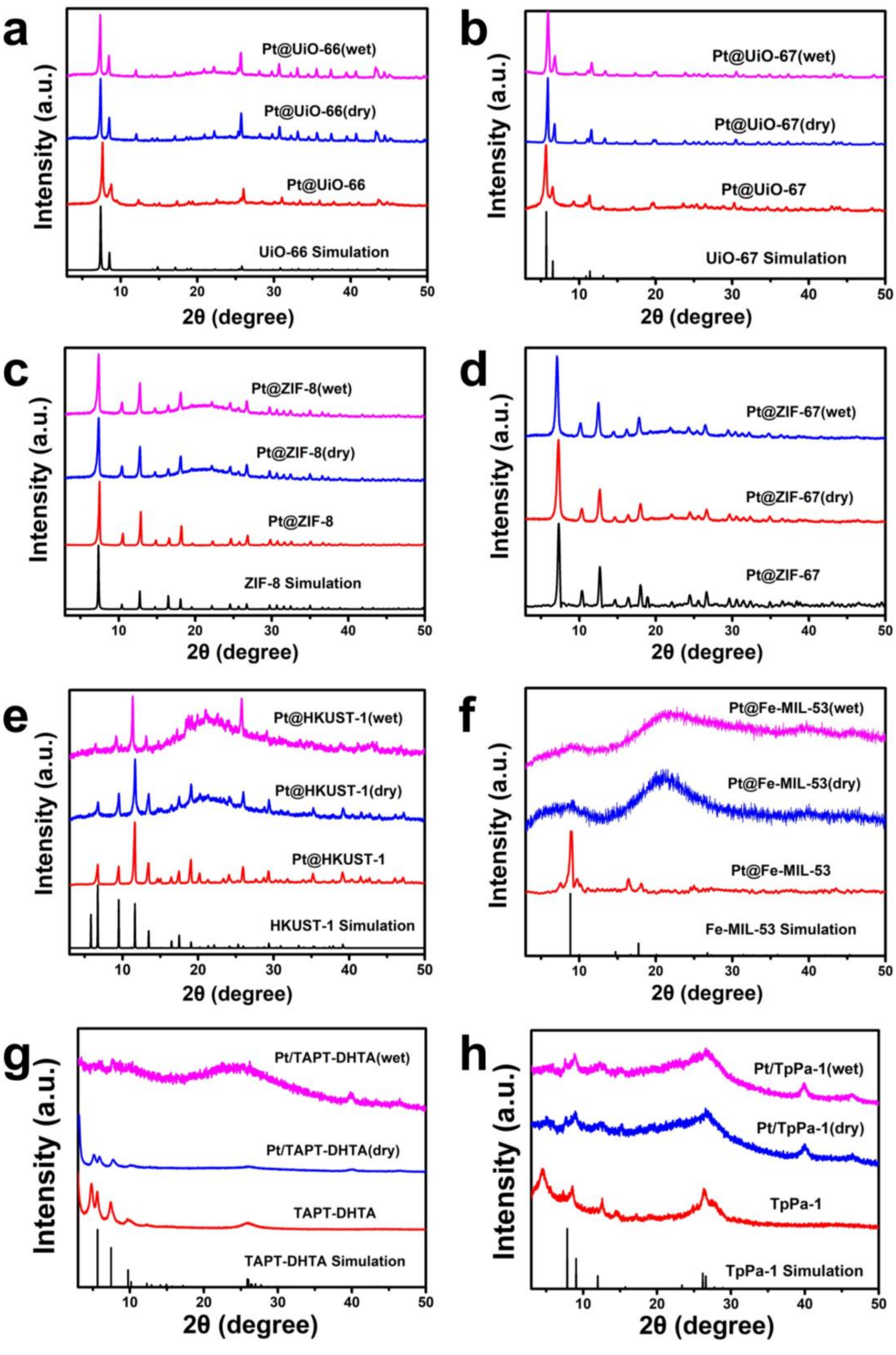

Supplementary Figure 39. PXRD patterns of (a) Pt@UiO-66, (b) Pt@UiO-67, (c) Pt@ZIF-8, (d) Pt@ZIF-67, (e) Pt@HKUST-1, (f) Pt@Fe-MIL-53, (g) Pt/TAPT-DHTA and (h) Pt/TpPa-1 after dry or wet H_2 treatment at 200 °C.

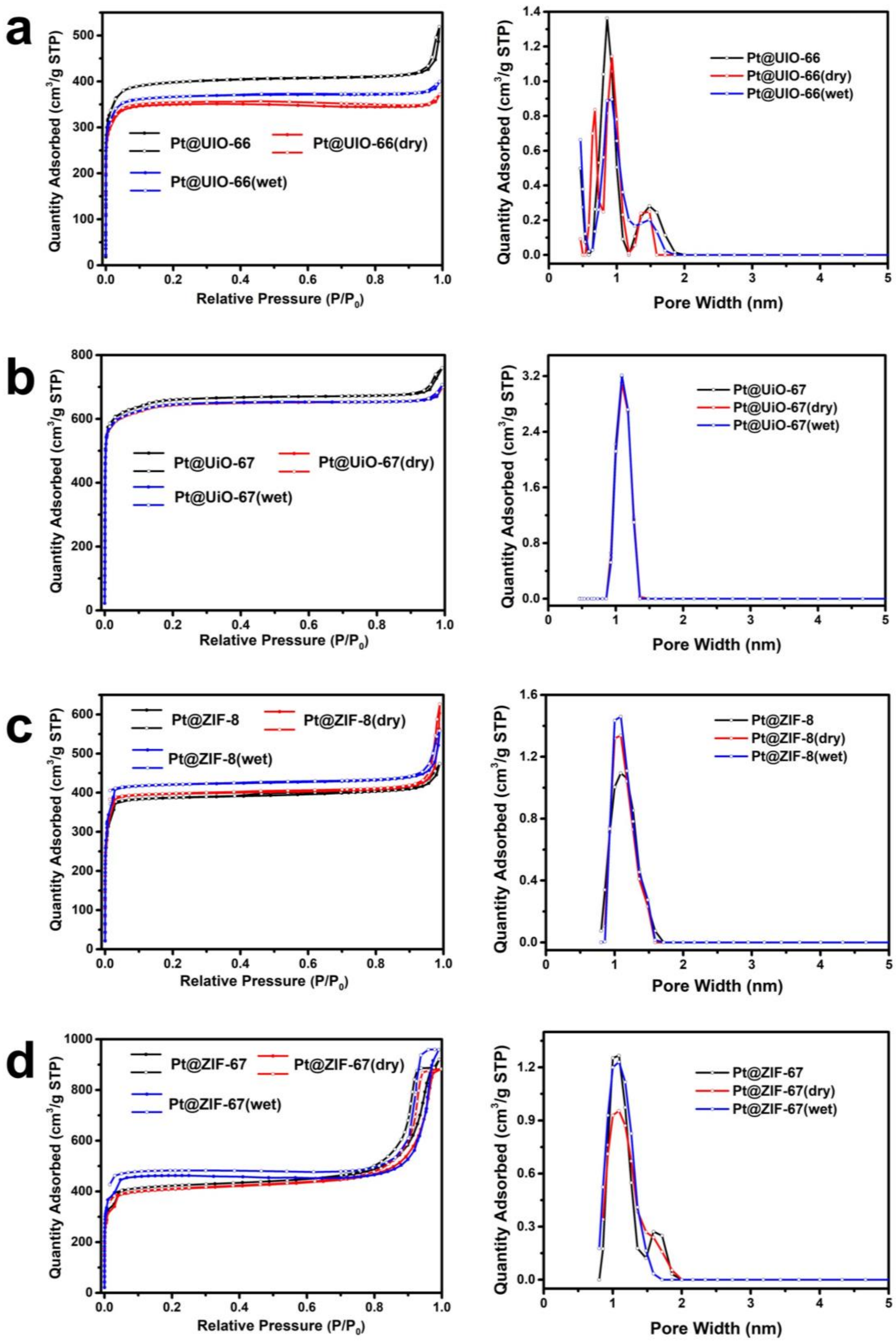

Supplementary Figure 40. Nitrogen adsorption–desorption isotherms and pore size distribution of (a) Pt@UiO-66, (b) Pt@UiO-67, (c) Pt@ZIF-8 and (d) Pt@ZIF-67 after dry or wet H_2 treatment at 200 °C.

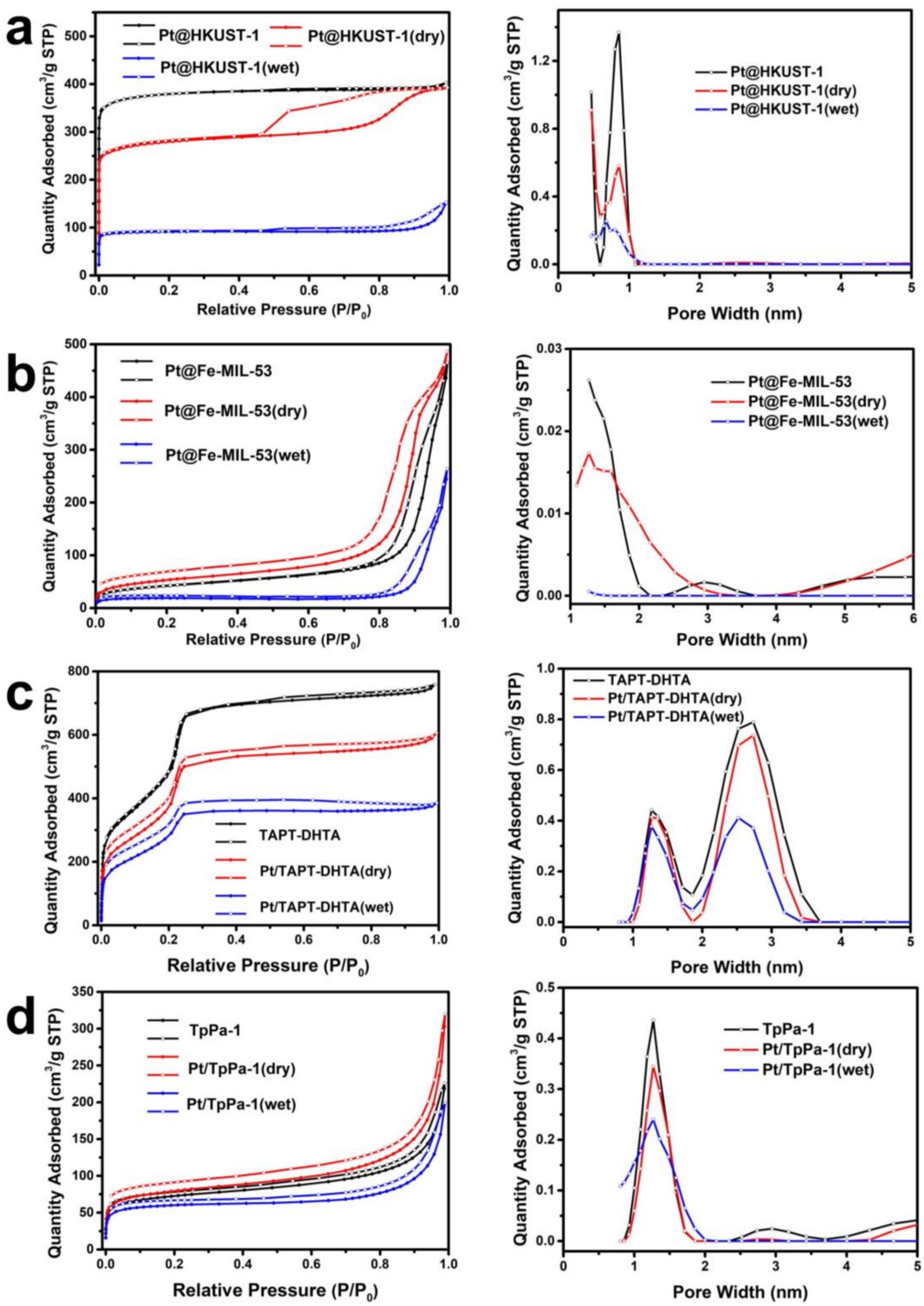

Supplementary Figure 41. Nitrogen adsorption–desorption isotherms and pore size distribution of (a) Pt@HKUST-1, (b) Pt@Fe-MIL-53, (c) Pt/TAPT-DHTA and (d) Pt/TaPa-1 after dry or wet H_2 treatment at 200 °C.

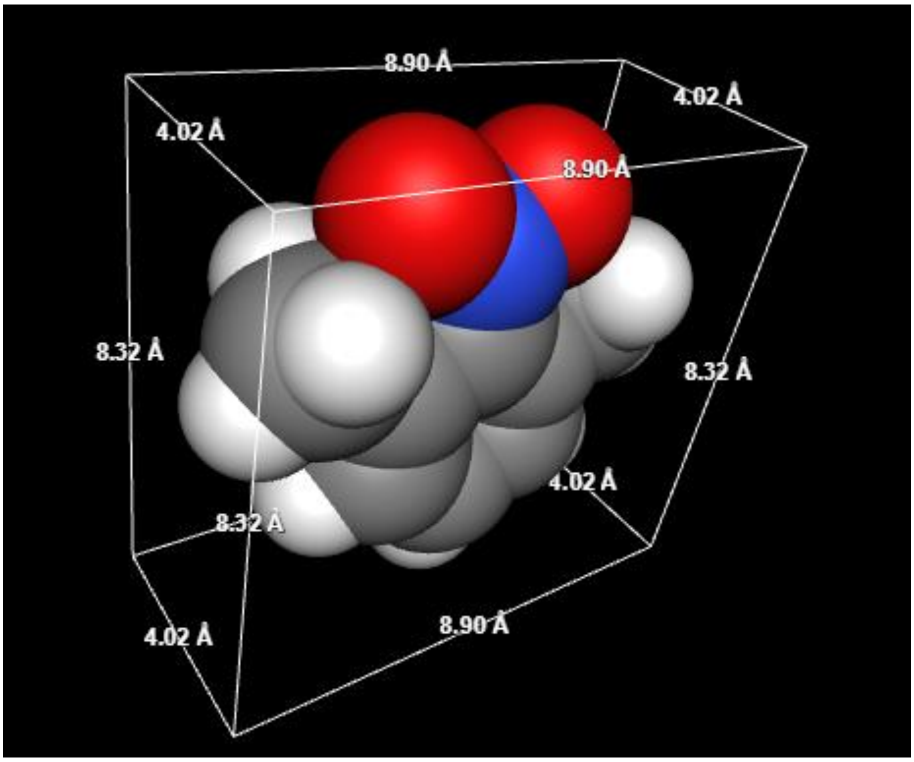

Supplementary Figure 42. The molecular size simulation of 2,6-dimethylnitrobenzene.

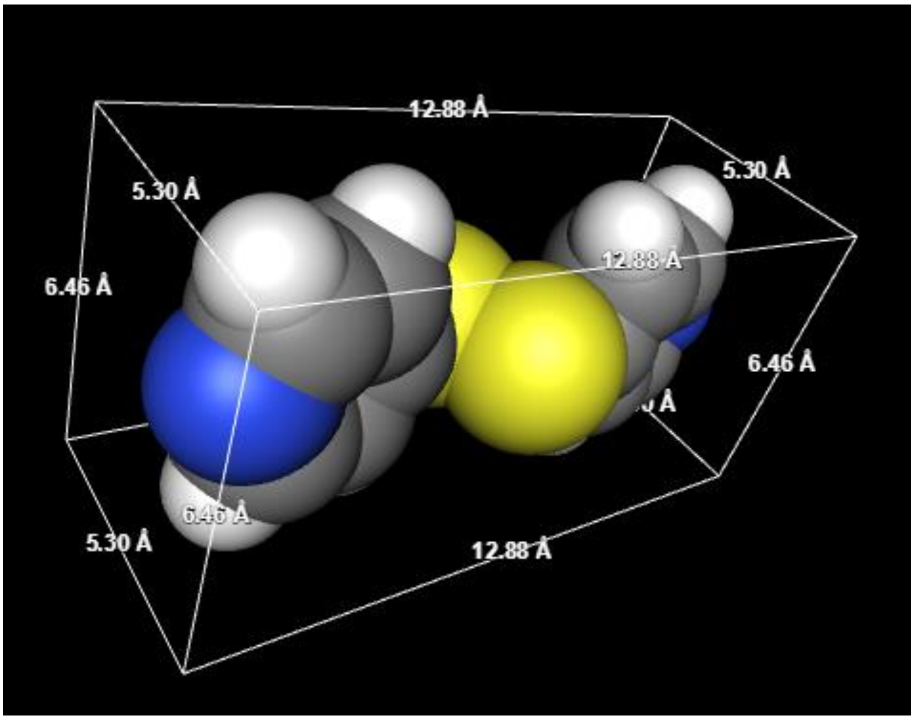

Supplementary Figure 43. The molecular size simulation of DTDP.

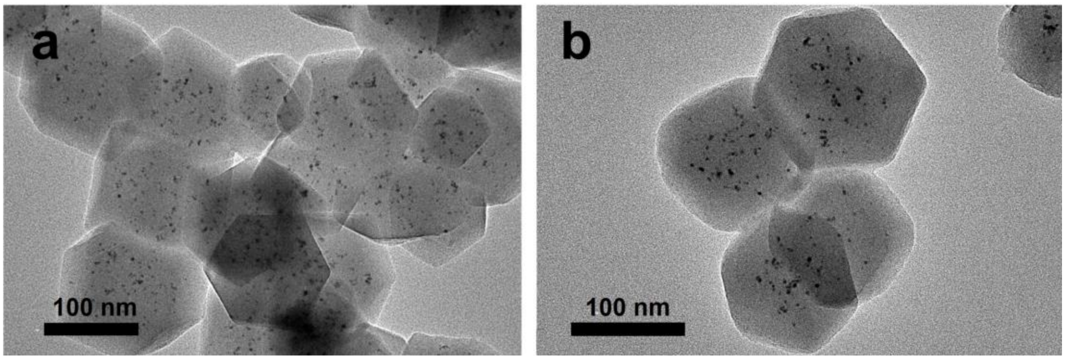

Supplementary Figure 44. TEM images of Pt@ZIF-8 (a) before and (b) after hydrogenation in wet H_2.

Supplementary References

1. Karim, W. et al. Catalyst support effects on hydrogen spillover. *Nature* **541**, 68–71 (2017).
